# Dentin reactions to caries are misinterpreted by histological “gold standards”

**DOI:** 10.12688/f1000research.3-13.v1

**Published:** 2014-01-16

**Authors:** Priscila Florentino Silva, Danilo Augusto de Holanda Ferreira, Kássia Regina Simões Meira, Franklin Delano Soares Forte, Ana Maria Barros Chaves, Frederico Barbosa de Sousa

**Affiliations:** 1 Department of Morphology, Health Science Center, Federal University of Paraiba, Cidade Universitária, Paraiba, 58051-900, João Pessoa, Brazil; 2 Laboratory of Microscopy and Biological Image, Health Sciences Center, Federal University of Paraiba, Cidade Universitária, Paraiba, 58051-900, João Pessoa, Brazil; 3 Department of Clinical and Social Dentistry, Health Sciences Center, Federal University of Paraiba, Cidade Universitária, Paraiba, 58051-900, João Pessoa, Brazil

## Abstract

Dentin reactions to caries, crucial for pathogenesis and for the determination of the severity of caries lesions, are believed to be reasonably detected by stereomicroscopy (SM) and polarized light microscopy in quinoline (PLMQ), but accuracies are not available. Here, stereomicroscopy of wet (SW) and dry (SD) ground sections of natural occlusal caries lesions resulted in moderate (0.7, for normal dentin) and low accuracies (< 0.6, for carious and sclerotic dentin) as validated by contrast-corrected microradiography. Accuracies of PLMQ were moderate for both normal (0.71) and carious dentin (0.71). The hypothesis that detection of dentin reactions by SM and PLMQ would be influenced by the contrast quality of micrographic images was rejected. Dentin reactions were scored by SW, SD, PLMQ, and three types of microradiographic images with varying contrast qualities and each technique was compared against the one that resulted in the highest number of scores for each dentin reaction. Large differences resulted, mainly related to the detection of sclerotic dentin by both SW and SD, and normal and carious dentin by PLMQ. It is concluded that contrast-corrected microradiography should be preferred as the gold standard and SM and PLMQ should be avoided, but the relationship of PLMQ with dentin mineralization deserves further investigation.

## Introduction

Dentin reactions to caries are crucial for the pathogenesis and severity determination of caries lesions. Since caries is mainly a demineralization process, the high ratio of X-ray absorbance between calcium and the chemical elements of the organic content ^1^, and the fact that the density of the mineral content is higher (more than 2 times) than that of the organic content ^2^, radiography with microscopic resolution (microradiography, MR) is considered as a highly reliable gold standard for detecting variations in dentin mineral content. Also widely accepted as gold standards for dentin reactions are stereomicroscopy (SM; commonly referred as histology) ^3, 4^ and (to a lesser extent) polarized light microscopy with quinoline as the immersion medium (PLMQ) ^5^. The acceptance of SW, the currently most used “gold standard”, is based on studies reporting opaque and translucent dentin under SM related to radiolucent and radiopaque dentin, respectively ^6– 9^. It is lacking, however, accuracy in numbers. To our knowledge, data regarding the accuracy of SM are available from only one study that included unerupted teeth and no data on translucent/sclerotic dentin ^10^. In addition, early studies with MR reported cases classified as translucent dentin by transmitted light microscopy (where the interaction of light with dentin is similar to that under SM) that were then classified as demineralized dentin by the use of MR ^11– 14^.

Currently the only evidence for the detection of dentin reactions by PLMQ is only qualitative ^5^. Thus, research into the accuracy of SM and PLMQ is needed. Regarding the use of MR as a gold standard, it must be considered that images of microradiographic plates taken using transmitted light microscopy are commonly biased by the effect of heterogeneous illumination, which is inversely proportional to the objective magnification ^15^. Heterogeneous illumination is expected to influence judgment of brightness ^16^ (a procedure required for diagnosis from MR images), possibly including bias when other techniques are validated using MR.

The aim of this study was three fold: to test the accuracy of both SM and PLMQ in detecting dentin reactions to natural caries; to test the hypothesis that elements of accuracy are influenced by the quality of the microradiographic image contrast; and to test the hypothesis that SM, PLMQ and MR (regardless of image contrast quality) detect dentin reactions equally.

## Materials and methods

### Diagnosis of occlusal caries lesions using ICDAS II

Forty three erupted third molars with various stages of natural occlusal caries were collected from volunteers who signed consent terms (as approved by the Ethical Committee of the Federal University of Paraiba; certificate of ethical appreciation number 4125.0.000.126-10). All teeth were gently cleaned with 1% hypochlorite solution (Vetec, Brazil), mounted in a wax base and surrounded by a rubber dam isolator prior to analysis of their occlusal surfaces using the ICDAS II scoring system ^17^. Before obtaining final ICDAS scores for analysis, examiners were calibrated using a sub-set of the whole sample. Thirty occlusal sites were scored by two calibrated examiners (Kappa’s intra-examiner’s scores of 0.9 and 0.89, and 0.85 for inter-examiner agreement), with a one week interval, in order to test intra- and inter-examiner reproducibilities. Final scores were those obtained by a consensus between examiners. In nine teeth, two sites on the occlusal surface were selected, yielding a total of 52 occlusal caries lesions.

### Ground section preparation

All teeth were cut longitudinally to their crowns (through their occlusal surfaces) using a diamond disc mounted (Kavo Sorensen, Brazil) in a low-speed handpiece under water irrigation, so that a section of the selected site with a given IDCAS score was obtained. All cuts were then ground using a customized metallic (brass) lapping jip and silicon carbide paper (granulations of 240–1200) under water irrigation to achieve a final thickness of ~100 ± 20 μm. Prepared ground sections (n = 52) were kept in a 0.02% sodium azide aqueous solution until examination.

### Calibration of examiners of ground sections and selection of histological sites

At each ground section, histological sites (area of ~150 μm × 150 μm) presenting suggestive signs of normal, carious, or sclerotic dentin were selected. All examinations of ground sections (SM of wet and dry samples, three types of MR images, and PLMQ) were performed by the same two examiners, whose intra and inter-examiner reproducibilities were determined (using Kappa’s statistics) from their scores of all histological sites (of all samples) from each technique obtained with a one week interval. Examiners agreed on the final scores by consulting with each other.

Histological sites were selected from the outer half of the dentin layer, including the area adjacent to the deepest enamel lesion; at least one dentin reaction type per sample was included where possible. Cases were included in the sample when two sites had the same type of dentin reaction detected by SM, while showing different types of dentin reactions when detected using MR. Thus, up to 6 histological sites were selected per sample, yielding a total sample size of 168 sites.

### Stereomicroscopy (SM)

SM (10× magnification) with reflected light was used to analyze ground sections under two conditions: wet (SW) and dried (SD; after exposure to 25ºC and 50% relative humidity for 2 hours). Temperature and relative humidity were measured just adjacent to the samples. Digital photomicrographs (digital camera Nikon D80) of wet and dried samples were obtained. Dentin reactions were scored as normal, carious discolored (white/yellow/brown), and translucent (“sclerotic”).

### Microradiography (MR)

All samples were mounted in a microradiographic plate (resolution of 2000 lines/mm; AGHD plates, Microchrome Technology, San Jose, USA) and exposed to X-rays in a PCBA Inspector (tungsten anode filtered with a 0.25 mm-thick beryllium window, GE, Germany) for 25 minutes using 40 keV and 0.25 mA. Digital photomicrographs of the microradiographic plate were obtained in a transmitted light microscope (2× objective) under different conditions:

Condition 1: using the condenser aligned according to the principles of Kohler illumination for low magnification objectives ^18^;

Condition 2: no condenser and using a light shaping filter (Luminit, USA) above the field diaphragm.

The possible scores for dentin reactions using MR were: normal dentin, demineralized (radiolucent) dentin, and hypermineralized (highly radiopaque; sclerotic) dentin. Digital images were analysed (using the freeware program ImageJ, NIH, USA) with the following contrast conditions:

Image obtained with aligned condenser, without any adjustment of brightness and contrast from ImageJ, and no light shaping filter (NFNBC image);

Image obtained with aligned condenser, no light shaping filter, but with adjustment of brightness and contrast from ImageJ (NFBC image);

Image obtained without a condenser, with both a light shaping filter and adjustment of brightness and contrast from ImageJ (FBC image).

Such conditions created an ordinary scale of heterogeneous illumination of the field of view. Images without light shaping filter (NF) and FBC images presented a Gaussian normalized light intensity (R ^2^ = 0.87 for both) across the field of view with heights of 0.13 and 0.05 (lower heterogeneity), respectively. Brightness and contrast adjustment (according to a consensus from both examiners) allowed this difference to be easily detected by the naked eye.

### Polarized light microscopy in quinoline (PLMQ)

Ground sections were dried at room temperature for 24 hours, immersed in quinoline (Vetec, Brazil) for 24 hours, and then were positioned with the dentin tubules at – 45º on the stage of a polarizing microscope (Axioskop, Carl Zeiss, Germany) equipped with a Red I filter, 2× objective, and digital camera (Nikon D80, Japan). Dentin reactions were scored as either negatively (carious) or positively birefringent (normal) ^5^. Since the technique of PLMQ is not intended to diagnosis dentin sclerosis ^5^, no diagnosis of sclerotic dentin was attempted. Color digital images were split into color channels using ImageJ, resulting in a sharp demarcation of negatively and positively birefringent areas.

### Accuracy of SM and PLMQ

We tested the accuracies of the SW, SD, and PLMQ techniques for detecting dentin reactions using the FBC MR image as the gold standard in all cases. Total positive (TP), total negative (TN), false positive (FP), and false negative values (FN) were obtained and used to calculate accuracy (AC) from:

$$AC=\frac{TN+TP}{TN+TP+FN+FP}\;\;\;\;\;(1)$$

### Effect of MR contrast on the detection of dentin reactions from SM and PLMQ

Positive (PPV) and negative predictive values (NPV) of each dentin reaction detected by SM were calculated using the following combinations:

1. SW × NFNBC;

2. SW × NFBC;

3. SW × FBC;

4. SD × NFNBC;

5. SD × NFBC;

6. SD × FBC.

The number of a given dentin reaction was that obtained from agreement from both the SM and MR images of each combination. Each SM technique was validated against all MR images types. The PPV was calculated from the ratio of the TP (determined by each MR image type) by the test outcome positive, and NPV was calculated from the ratio of the TN (determined by each MR image type) by the test outcome negative.

In order to test whether PPV and NPV were altered by dentin hydration and or MR contrast, combinations 1–5 were compared with combination 6 (considered as the gold standard combination) and Cohen’s effect size for proportions ( *h*) was calculated from ^19^:

$$h=\phi_{x}-\phi_{y}\;\;\;\;\;(2)$$

and

$$\phi =2\cdot \arcsin\sqrt{P}\;\;\;\;\;(3)$$

Where *ϕ _x_* and *ϕ _y_* are the arcsines (in radians) of the proportions “P” (PPV or NPV), of a given dentin reaction from the test combination (1–5) and gold standard combination, respectively. The same test was performed with PLMQ, using:

1. PLMQ × NFNBC;

2. PLMQ × NFBC;

3. PLMQ × FBC.

Equation (2) was used to calculate the difference between combinations 7–8 and combination 9 (gold standard).

### Effect of “temporary gold standard” on the proportions of dentin reactions

Dentin reactions were summed separately as detected from each of the six techniques tested and converted to proportions in relation to the number of sites detected by the technique that yielded the highest number (this later ascribed as the “temporary gold standard”). Equation (2) was used to calculate the difference between the “temporary gold standard” proportion ( *ϕ _x_*) and the test proportion ( *ϕ _y_*).

### Correlation between translucency and radiopacity

We tested the hypothesis that translucency under SM was related to the radiopacity in dentin. Five ground sections (with ICDAS scores ranging from 0 to 2) were microradiographed in a digital X-ray machine (Faxitron model MX20, Tucson, USA; tungsten anode filtered with a 0.25 mm thick beryllium window) using 20 keV, 0.3 mA and digitally photomicrographed under SM (with dry dentin; SD) with a dark background created with a neutral filter. Under SM, samples presented normal and translucent dentin sites only. Faxitron images had an almost flat illumination across the field of view (Gaussian fit: R ^2^ = -0.0004 and height of 0.028; and linear fit: R ^2^ = 0.18). Eighty histological sites (16/sample) were selected and gray levels were measured on both SM and Faxitron digital images using image analysis software (ImageJ, NIH, USA). The maximum intensity of both image histograms was lower than 80% of maximum intensity allowed. Translucency and radiopacity were measured by:

$$\frac{I_{ext}-\Delta I}{I_{ext}}\;\;\;\;\;(4)$$

Where ΔI is the difference (always converted to a positive value) between the intensity of the histological site and the maximum intensity of image histogram (I _ext_; maximum intensity for radiopacity, and minimum intensity for translucency). Translucency and radiopacity were normalized and then analyzed using Pearson product-moment correlation coefficient.

## Results

The occlusal caries ICDAS scores for the samples analysed were: 10 with score 0, 2 with score 1, 23 with score 2, 10 with score 3, 1 with score 4, 4 with score 5, and 2 with score 6. For microscopy, intra-examiner agreements were (examiner 1/examiner 2): 0.896/0.914 (SW), 0.952/0.944 (SD), 0.909/0.899 (NFNBC), 0.90/0.91 (NFBC), and 0.919/0.898 (FBC). The inter-examiner agreements were 0.914 (SW), 0.953 (SD), 0.918 (NFNBC), 0.949 (NFBC), and 0.979 (FBC).

**Figure 1.**
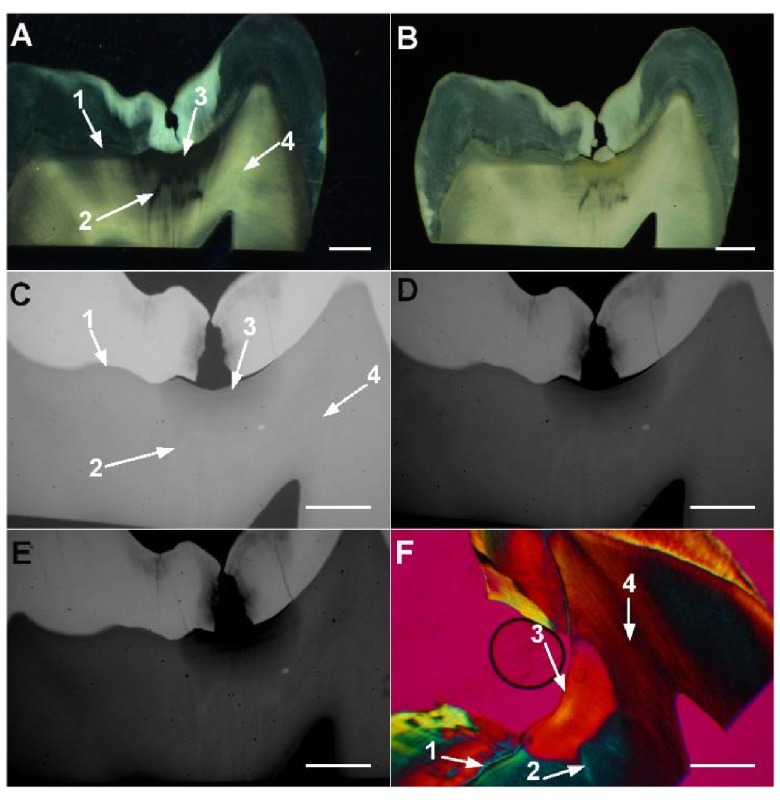
Typical aspects of dentin reactions in selected histological sites (labeled 1–4 with arrows) of natural occlusal caries (ICDAS II score 2). Translucent dentin is shown in sites 1–3 from the SW image ( **A**) and in sites 1 and 2 from the SD image ( **B**). Site 1 appears as demineralized dentin in all types of MR images and as negatively birefringent (“carious dentin”) in PLMQ ( **F**). Site 2 appears as normal dentin in the NFNBC image ( **C**), as hypermineralized (sclerotic) in both NFBC ( **D**) and FBC ( **E**) images, and as negatively birefringent in PLMQ. Site 3 appears as carious (yellowish) dentin in the SD image ( **B**), as demineralized dentin in all types of MR images, and as positively birefringent (“normal”) in PLMQ. The appearance of site 4 is of normal dentin in all images. Bars = 1 mm.

Typical aspects of normal, carious and sclerotic/translucent histological points under the six techniques tested here are shown in Figure 1. The number of dentin reactions detected varied among techniques ( Table 1). Detection of normal dentin had a moderate accuracy for both SW (0.7976) and SD (0.7976), while both techniques presented a low accuracy for detecting carious (0.5952 for SW; 0.631 for SD) and sclerotic dentin (0.5833 for both SW and SD). Regarding PLMQ, the accuracy was moderate for both carious (0.7092) and normal dentin (0.7902).

**Table 1.** Number of dentin reactions detected from each technique.

*Technique*	*Dentin reaction*		
	*Normal*	*Carious*	*Sclerotic/translucent*
SW	59	31	78
SD	49	37	82
NFNBC	77	76	18
NFBC	66	84	15
FBC	59	89	20
PLMQ	24	129	---

PPV and NPV values of SW, SD, and PLMQ for dentin reactions from combinations 1–9 are shown in Table 2. For normal dentin, SW and SD had moderate PPV and NPV, but for carious and sclerotic dentin they ranged from very low to high values. This later behavior was observed for PLMQ from all combinations.

**Figure 2.**
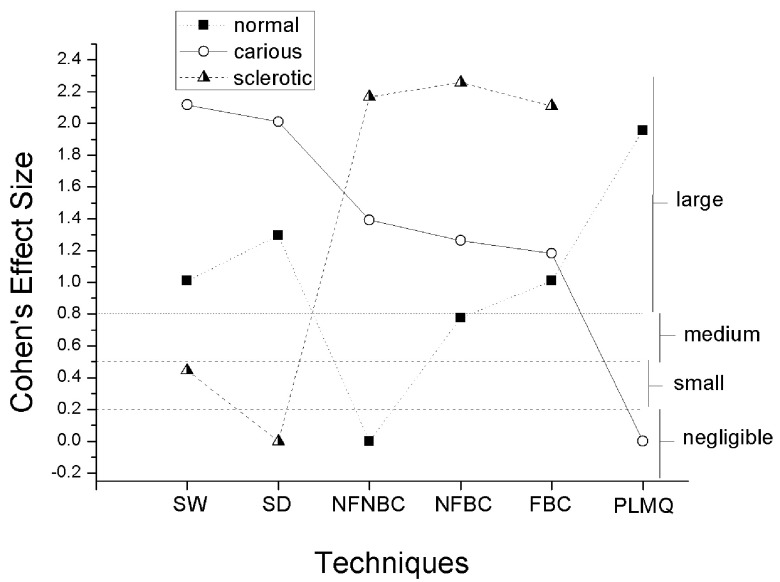
Effect sizes of comparisons between the “temporary gold standard” and other techniques on the detection of normal (black square + dashed line), carious (open circle + continuous line), and sclerotic (half-filled triangles + short dashed line) dentin. Labels on the right are magnitudes of effect size in relation to the “temporary gold standard” only.

NFNBC, PLMQ, and SD were the “temporary gold standards” for normal, carious, and sclerotic dentin, respectively ( Figure 2). Regarding both normal and carious dentin, all comparisons of the “temporary gold standard” with other techniques resulted in large effect sizes. For sclerotic dentin, SM techniques had a small difference, while large effects sizes were measured for comparisons with MR techniques ( Figure 2).

**Table 2.** PPV and NPV of SM and PLMQ for dentin reactions from all combinations of test and gold standard outcomes.

*PPV and NPV*	*Dentin reactions*		
	*Normal*	*Carious*	*Sclerotic*
**SW × NFNBC**			
PPV	0.83	0.77	0.13
NPV	0.74	0.62	0.94
**SW × NFBC**			
PPV	0.78	0.77	0.17
NPV	0.82	0.56	0.94
**SW × FBC**			
PPV	0.71	0.84	0.18
NPV	0.84	0.54	0.93
**SD × NFNBC**			
PPV	0.86	0.78	0.15
NPV	0.71	0.64	0.97
**SD × NFBC**			
PPV	0.84	0.86	0.18
NPV	0.79	0.60	0.97
**SD × FBC**			
PPV	0.76	0.86	0.20
NPV	0.82	0.56	0.95
**PLMQ × NFNBC**			
PPV	0.26	0.94	---
NPV	0.94	0.26	---
**PLMQ × NFBC**			
PPV	0.31	0.95	---
NPV	0.95	0.31	---
**PLMQ × FBC**			
PPV	0.33	0.95	---
NPV	0.95	0.33	---

**Figure 3.**
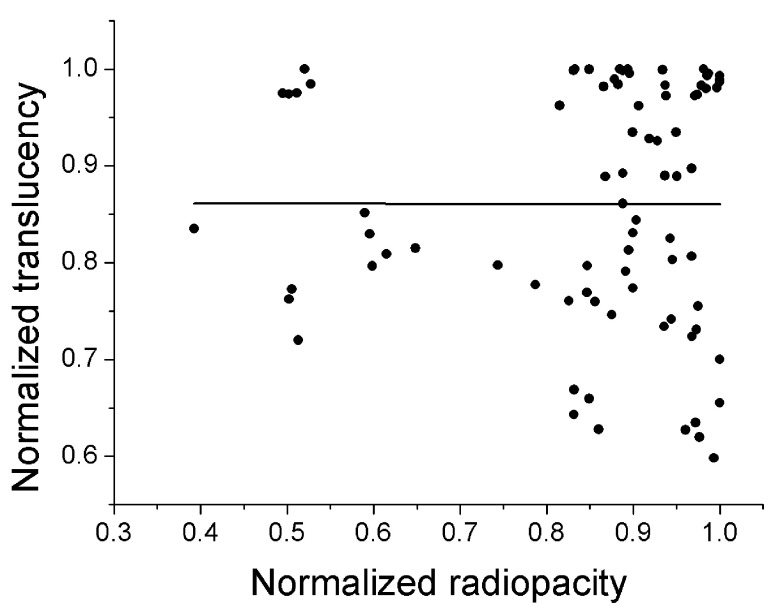
Plot of normalized radiopacity against normalized translucency showing no correlation (black line = Pearson’s correlation fit; R ^2^ = -0.013). N = 80 histological sites.

 The effects sizes of MR image contrast on the PPV and NPV of different combinations of SM and MR was negligible ( Table 3). The corresponding values for PLMQ were low: (i) for normal dentin, effect sizes of 0.154 (PLMQ×NFNBC) and 0.043 (PLMQ×NFBC) for PPV, and 0.044 (PLMQ×NFNBC) and 0.0 (PLMQ×NFBC) for NPV; and (ii) for carious dentin, effect sizes of 0.044 (PLMQ×NFNBC) and 0.0 (PLMQ×NFBC) for PPV, and 0.154 (PLMQ×NFNBC) and 0.043 (PLMQ×NFBC) for NPV. Analysis of the relationship between translucency and radiopacity of dentin showed that they were not correlated ( Figure 3).

**Table 3.** Comparisons (effect sizes) between tests (1–5) and gold standard (6) combinations of stereomicroscopy and microradiography on PPV and NPV of dentin reactions.

Parameter	Combinations				
	SW×NFNBC	SW×NFBC	SW×FBC	SD×NFNBC	SD×NFBC
**Normal dentin**					
*PPV*	-0.174	-0.048	0.113	-0.257	-0.201
NPV	0.194	0.000	-0.053	0.261	0.076
**Carious dentin**					
PPV	0.233	0.233	0.056	0.209	0.000
NPV	-0.122	0.000	0.040	-0.164	-0.081
**Sclerotic dentin**					
PPV	0.190	0.077	0.051	0.132	0.051
NPV	0.044	0.044	0.085	-0.103	-0.103

Dentin reactions detected from SM and PLMQ Data file 1: Radiopacity versus translucency of dentin
This file contains normalized data on translucency and radiopacity of histological sites from five ground sections.
Data file 2: SW, SD, NFNBC, NFBC, FBC and PLMQ scores per histological site
ICDAS scores for each carious lesion and the SW, SD, NFNBC, NFBC, FBC, and PLMQ scores for each histological site of each ground section. Codes for SM scores: 1 (normal), 2 (carious), and 3 (translucent). Codes for MR scores: 1 (normal), 2 (demineralised), and 3 (hypermineralized). Codes for PLMQ scores: 1 (positive birefringence) and 2 (negative birefringence).

## Discussion

Here we used Cohen’s effect size ^19^ to test differences between outcomes of a given gold standard and a test technique. Effect sizes > 0.5 mean that differences are higher than the acceptable amount to neglect them. In some cases, more than one section per tooth was analyzed. There is conflicting data on whether demineralization can spread laterally to the main dentinal tubules (close to the enamel-dentin junction) or not in non-cavitated carious lesions ^20, 21^. If lateral spread does occur, dentin demineralization and sclerosis might occur at random in different sections of a same lesion. In addition, two sites located at different fossae on the same occlusal surface are expected to present independent dentin reactions. This is why we considered more than one section per tooth as independent samples.

This study showed that SM has a low accuracy for detecting carious and sclerotic dentin. This agrees with early qualitative reports showing that translucent dentin can be either caries or sclerosis ^11– 14^. The explanation is that translucency is related to discontinuities in refractive indexes ^22^, but is not necessarily related to radiopacity ( Figure 3). When we tested the assumption that any technique is likely to measure dentin reactions equally, pronounced differences were obtained for all dentin reactions, with the most pronounced difference being that between SM and the other techniques with regard to sclerotic dentin ( Figure 2). PLMQ, another technique regarded (without evidence) to be useful for detecting normal and carious dentin ^5^, showed the most pronounced differences for the detection of normal and carious dentin. To our knowledge, there are no data explaining the relationship between birefringence and dentin mineral content, and this gap impedes further consideration of using PLMQ for detecting dentin reactions. Its use should be avoided until its relationship with dentin mineral content is clarified. The large effect sizes obtained when PLMQ was the temporary gold standard and moderate accuracies of PLMQ are intriguing, and worth further investigation.

Previous studies that have reported qualitative evidence of translucent dentin under SM related to radiopaque dentin ^6– 9^, and have been cited as the basis for regarding SM as the gold standard ^23^, performed their analysis with images showing more than 2 mm × 2 mm of the tooth crown. Such a field of view size can only be obtained from low magnification objectives ^24^. Heterogeneous illumination of the field of view in light microscopy images is inversely related to objective magnification ^15, 24^. Thus, heterogeneous illumination may have been a common factor in their analyses, and most probably our NF MR images (NFNBC and NFBC) are the ones that more closely resemble the images obtained in these older studies. The hypothesis that MR image contrast (influenced by heterogeneous illumination) could explain variations in the PPV and NPV of dentin reactions was rejected ( Table 2). Thus, our data suggest that the reason why scientists misinterpreted dentin aspects under SM is not because they used poorly contrasted microradiographs, but probably due to a result of a lack of accuracy. The assumption, now shown quantitatively to be wrong, that translucent dentin under SM always represents sclerotic or non-carious dentin is currently highly influential in the selection of normal dentin, and in the determination of the onset ^25– 27^ and extent (perhaps the most important) of the carious process in dentin. An indication of such influency is that currently, considering most popular ^23, 28^ and recent ^21^ textbooks, nearly no one is trained in cariology and dental histology without being presented to SM images showing translucent dentin interpreted as sclerotic dentin.

Some implications of our study can be explained by using the example of a SM image of a section of a carious tooth. On the occlusal surface, opaque enamel in the outer one third of the enamel layer combined with translucent dentin in the outer 300 microns of the dentin layer are interpreted as an indication that the carious lesion is confined to the outer enamel and that the dentin has already reacted to it by producing sclerotic dentin. Our results and previous evidence ^11– 14^ show that this translucent dentin might actually be carious demineralization. This alters the interpretation of the lesion depth and also the understanding of how carious demineralization propagates in the hard dental tissues. In this context, SM data without *quantitative* evidence of mineral content through the carious lesion might be misleading. In addition, or alternatively, it might be misleading to ignore the possibility that caries formation is a result of two relatively independent events: initial acid infiltration and late demineralization, as shown experimentally ^29^. Cariogenic acid could infiltrate (from the tooth surface) down to the inner enamel and outer dentin prior to demineralization of the entire enamel layer located more externally. Acid could infiltrate into the tooth crown following enamel sheaths (the main pathways for transport of materials in enamel) ^30^, which are large nanochannels at the boundaries of enamel prisms, under the influence of an osmotic gradient ^31^ created by the higher organic content found in inner enamel ^32^. Demineralization at the early stages of caries lesion formation could take place at two locations: (i) the enamel surface, and (ii), a bit later than at the enamel surface, the region near the enamel-dentin junction (involving both enamel and dentin). At the enamel surface because it is closer to the source of cariogenic acid. At the region near the enamel-dentin junction because there the osmotic gradient would be minimized so that acid could move more slowly and find more favourable conditions to diffuse to the surfaces of the mineral crystallites surrounding the main pathways in enamel and dentin.

New optical techniques for caries diagnosis have been validated with SM only ^4, 33^. The assumption behind such validation is that SM has an acceptable accuracy for detecting dentin demineralization and sclerosis. Our results show that the probability of a correct diagnosis of both carious and sclerotic dentin using SM is low. Clinical visual caries diagnostic systems validated primarily by SM ^34, 35^ should have their validation tested using MR corrected with regard to heterogeneous illumination. The nature of dental caries should be studied without the bias related to the aspect of dentin under SM.

We conclude that, except for normal dentin, SM has low accuracy for detecting dentin reactions related to caries, and SM and PLMQ accuracies are not influenced by the quality of MR image contrast. FBC microradiographic images should be preferably used as the gold standard for judging dentin reactions.

## Data availability

*Figshare:* Dentin reactions detected from SM and PLMQ, doi: 10.6084/m9.figshare.895737 ^36^
