# Supplementary material for: Dentin reactions to caries are misinterpreted by histological “gold standards”
Source: F1000Res. 2014 Jan 16;3:13. [Version 1] doi: 10.12688/f1000research.3-13.v1 (PMC4240241; doi:10.12688/f1000research.3-13.v1)
Supplement: Peer review history

# Peer review: Referee response for version 1

**DOI:** 10.5256/f1000research.3418.r6314

**Published:** 2014-11-13

**Authors:** Chris Deery

**Affiliations:** 1 The School of Clinical Dentistry, The University of Sheffield, Sheffield, UK

This is an interesting paper which asks some interesting questions. The methodology is well described.

The title is appropriate. The abstract should be expanded to make the findings clearer. The main text has a minor problem with the use of abbreviations, this makes the paper hard to read particularly pages 5 and 6.

In the introduction there is no mention of other techniques such as micro-CT, ICDAS is just ICDAS not ICDAS II.

Page 4: accuracy is not the best method to use to assess the results, as it tends to inflate the results.

I think on page 4 1.SWxNFNBC etc could be better explained.

The Kappa results on page 5 paragraph 3 might be better presented as a table.

The authors should make a stronger explanation of why they believe microradiology is in fact more valid. Perhaps the problems they suggest are present with steriomicroscopy due to training or interpretation issues. They do not mention any training only calibration, these authors must be aware that reproducibility is very different to validity. This issue should be addressed.

I have read this submission. I believe that I have an appropriate level of expertise to confirm that it is of an acceptable scientific standard.

# Peer review: Referee response for version 1

**DOI:** 10.5256/f1000research.3418.r6313

**Published:** 2014-11-11

**Authors:** Mariana M. Braga

**Affiliations:** 1 Department of Pediatric Dentistry, University of São Paulo, São Paulo, Brazil

The present paper concerns a relevant topic related to dentin reactions to dental caries lesions. However, the authors’ intention, testing accuracies of methods for detecting these reactions, could be valorized/stressed in this paper. I believe that it is important to readers to know how important is to go beyond in this topic. In addition, considering the rationale, the authors stated that “only evidence for the detection of dentin reactions is qualitative”. I would recommend taking care to avoid misunderstandings, since the methods tested were qualitative, but I suppose they are looking for figures about accuracy of these methods. I suggest clarifying that. Another point to be clarified is the use of microradiography as a reference method. In the text, authors mentioned that “radiography with microscopic resolution (microradiography, MR) is considered as highly reliable gold standard for detecting variations in dentin mineral content." Is that an authors’ affirmation or is based on a previous background? Another aspect regarding reference methods is that the authors proposed to use the microradiography as gold standard, but, actually, they believed the microradiography was similar to other methods tested (according to the 3 ^rd^ objective). Why not choose an actual quantitative method for that? The rationale for that could be better developed in the introduction. Was that the idea of authors initially or was that a concept formulated *a posteriori *(after discussing and analyzing present findings)? Maybe, showing the reasons why opting for testing methods using a reference you “ suspect”  that is similar to other methods . I suggest these ideas could be explored in the Introduction.

Concerning methodology, one point remained unclear for this reviewer. In the paragraph *“Histological sites were selected from the outer half of the dentin layer, including the area adjacent to the deepest enamel lesion; at least one dentin reaction type per sample was included where possible. Cases were included in the sample when two sites had the same type of dentin reaction detected by SM, while showing different types of dentin reactions when detected using MR. Thus, up to 6 histological sites were selected per sample, yielding a total sample size of 168 sites."* it seems that only specimens in which there was some dentin reaction were selected. Is that true? If no, please, clarify that in the text and do not consider the next part. If yes, I believe this step could have led to spectrum of disease bias and selection bias, as well. If you try to detect the dentin reaction in a pool of histological sites, you tend to have a different performance than if you only selected sites in which the reaction is present. In addition, if you examined the teeth by one of the methods tested subsequently, you tend to favor this method in further examinations (because the selection was based on that). Based on these points, if you really selected only the sites with dentin reactions, I suggest including some information about those points in the Discussion.

Regarding the results, I suggest including the confidence intervals to facilitate the comprehension of possible overlapping of marginal values. Besides, I would include some discussion about the reference standard used and the relevance of detecting this level of demineralization in clinical setting or other applications. If the purpose is choosing the best treatment to the patients, some caution should be had in inferring data about very precise methods for demineralization quantification (sometimes these differences could lead to no difference in clinical approach to be adopted). But, certainly, other purposes exist and the investigation is valid for them. Moreover, I would explore more deeply how the figures showed for methods performance impact on inferences (e.g. which is the contribution of testing predictive values instead of showing only accuracy. Considering these points, I suggest some caution in writing the title.

The authors stated that “If lateral spread does occur, dentin demineralization and sclerosis might occur at random in different sections of a same lesion. In addition, two sites located at different fossae on the same occlusal surface are expected to present independent dentin reactions. This is why we considered more than one section per tooth as independent samples”. I believe that it could be true, but, to reinforce this assertive, I suggest testing the influence of the sampling in the present results or using a statistical approach that consider the clustering effect for the specimens evaluated.

Finally, I would consider separating the analysis for dentin reactions related to caries lesions. Despite being reactions in different ways (desmineralization vs. hypermineralization), caries lesion formation and sclerotic dentin formation are not actually opposite actions. They involve different mechanisms of pathogenesis and not only the quantity of minerals. That is why I believe the purpose of identifying each one of these alterations has its importance independently of the other. This separation, in my point of view, may help the authors in explaining the findings of this study. Additionally, based on this observation, I would also suggest reinforcing that in other parts of this paper.

I have read this submission. I believe that I have an appropriate level of expertise to confirm that it is of an acceptable scientific standard, however I have significant reservations, as outlined above.

# Peer review: Referee response for version 1

**DOI:** 10.5256/f1000research.3418.r5982

**Published:** 2014-09-09

**Authors:** Vera Mendes Soviero

**Affiliations:** 1 Department of Preventive and Community Dentistry, State University of Rio de Janeiro, Rio de Janeiro, Brazil

The manuscript is related to an interesting and relevant issue. The analysis of different gold standards for caries assessment than stereomicroscopy is of great interest, as we all know that the “histological gold standard” has limitations. However, in my view, certain points need to be clarified. I have carefully revised the manuscript and I hope the comments below are helpful to improve the quality of its final version.

**ABSTRACT**

I do recommend rewriting the abstract. It should be structured with Aim, Methods, Results and Conclusion in this order. It is not necessary to mention the titles, but it is important to follow this sequence. The aims are not mentioned in the abstract and results are shown before methodology.

**INTRODUCTION**

***1 ^st^ paragraph, lines 6 to 8***

*“… radiography with microscopic resolution (MR) is considered as a highly reliable gold standard for detecting variations in dentin mineral content.”*

I miss a reference for this statement. If MR was not validated previously for the detection and assessment of dentin caries or dentin reactions to caries, the aim of the study should be changed. Instead of testing the accuracy of stereomicroscopy against MR, it should be testing the accuracy of MR against stereomicroscopy. Reference number 10 (Hintze et al. 1995) concluded that SM was more reliable than MR. References number 11 to 14 (Applebaum et al. 1933; Applebaum 1935; Gottlieb 1946; Gottlieb et al. 1946) don’t seem to refer to validation studies.

**AIM**

First aim: how to test the accuracy of SM if it is established as gold standard?

Second aim: I didn’t understand. Please, explain better what is “elements of accuracy”.

Third aim: In my view, the third aim summarizes everything.

**MATERIALS AND METHODS**

***Calibration of examiners of ground sections and selection of histological sites***

Please, clarify better how calibration was done. What means *“Examiner agreed on the final scores by consulting with each other” *? Does it mean that examinations were not done independently?

Please, clarify the following sentence: *“… included in the sample when two sites had the same type of dentin reactions detected by SM, while showing different types of dentin reactions when detected using MR.” *It is not clear for me. If MR had to be different from SM, the results would be completely biased by this “selection criterion”. Or not?

**RESULTS**

***Tables in general***

Abbreviations should be explained using notes underneath the tables.

***Table 1***

Carious and sclerotic dentin was observed in all samples. How do you explain it considering that 10 teeth were scored visually as ICDAS 0? The sums in table 1 vary from 153 (PLMQ) to 171 (NFNBC). Why?

***Figure1***

The legend is not clear. Figures are not explained following the sequence of numbers. The arrows should be seen in all pictures.

***Table 2***

The title is not clear enough. I suggest writing in the title that microradiography was used as gold standard.

***Table 3***

*In the title, what means (1-5) and (6)? Please, clarify the title.*

I have read this submission. I believe that I have an appropriate level of expertise to confirm that it is of an acceptable scientific standard, however I have significant reservations, as outlined above.

# Response

**Published:** 2014-09-30

**Authors:** Frederico Sousa

**Affiliations:** Federal University of Paraiba, Brazil

The authors would like to thank the reviewer for her valuable technical comments, which will certainly contribute to improve the quality of the paper. The answers provided below are listed following the sequence of the reviewer comments and were prepared in common agreement among authors. Shortly we will publish a revised version of the article including the changes resulted from this review.

**Answer to comment on abstract: **We agree with the referee. The abstract will be changed in the revised version and here follows how it will be written:

"Dentin reactions to caries, crucial for pathogenesis and for the determination of the severity of caries lesions, are believed to be reasonably detected by stereomicroscopy (SM) and polarized light microscopy in quinoline (PLMQ), but accuracies are lacking. We aimed at testing (i) accuracies of SM and PLMQ, (ii) the effect of image contrast of microradiography (MR) on accuracy, and (ii) the hypothesis that SM, PLMQ and MR (regardless image contrast) equally detect dentin reactions. Ground sections (n = 63) of natural occlusal caries (ICDAS scores 0 to 6) were analyzed using MR as the gold standard. SM of wet and dry ground sections of natural occlusal caries lesions resulted in moderate (0.7, for normal dentin) and low accuracies (< 0.6, for carious and sclerotic dentin) as validated by contrast-corrected MR. Accuracies of PLMQ were moderate for both normal (0.71) and carious dentin (0.71). The main differences were related to the detection of sclerotic dentin by both SW and SD, and normal and carious dentin by PLMQ. The hypothesis that detection of dentin reactions by SM and PLMQ would be influenced by the contrast quality of MR images was rejected. In conclusion, contrast-corrected MR should be preferred as the gold standard and SM and PLMQ should be avoided, but the relationship of PLMQ with dentin mineralization deserves further investigation." 

**Answer to comments on introduction:** The following papers can be used as references for the statement that microradiography is considered as a reliable gold standard for variations in dentin mineral content: Ten Bosch & Angmar-Månsson (1991); and Arends *et al. *(1997). However, the fundamental reason why microradiography is a reliable gold standard lies on what was stated in the first six lines of the Introduction. Here, we explain those reasons more clearly. Basically (and this applies for the analysis of mineralized tissues by radiography in general), from the components of dentin (mineral, organic matter and water), the mineral content absorbs X-rays more intensively than the other components. The basic parameter is the linear attenuation coefficient (LAC) for X-rays, which is the product of the mass attenuation coefficient (MAC) by the density of the unit cell. Information on MAC and LAC (corresponding to X-ray peak of 24 keV, which is the emission peak emitted by the X-ray source used here operating at 40 kV and 0.25 mA) for both the mineral and the organic components of dentin are listed below:

1. For the mineral component: with the unity cell’s empirical formula Ca _8.856_Mg _0.088_Na _0.292_K _0.010_P _5.592_Cl _0.078_O _25.561_H _3.222_C _0.457_ (reference 2), the MAC is 3.83 cm ^2^•g ^-1^, the density is 2.99 g•cm ^-3^ (reference 2), and the LAC is 11.45 cm ^-1^.
2. For the organic component, represented by collagen: with the unity cell’s empirical formula C _12_H _24_N _3_O _4_ ( Omokanwaye *et al.*, 2010), the MAC is 0.41 cm ^2^•g ^-1^, the density is 1.19 g•cm ^-3^ ( Gautieri *et al.*, 2011), and the LAC is 0.49 cm ^-1^.

MACs for the chemical elements of the empirical formulas listed above can be found in reference 1. Gray levels of dentin in MR are the result of the product of the volume of each component by its respective LAC, and the sum of all products is multiplied by sample thickness. Thus, as mineral volume decreases in dentin, gray levels decrease (i.e., dentin becomes less radiopaque), and vice-versa.

With light scattering, which governs the aspect of dentin under SM, is different. By matching the refractive index of an immersion liquid with the refractive index of dentin light scattering is reduced, rendering the tissue more translucent without modifying tissue composition ( Fried *et al*., 1995). In addition, visible light scattering in dentin is highly influenced by the density ( Zijp & Bosch, 1993; Kienle *et al*., 2003) and angulations (Kienle *et al*., 2003) of dentinal tubules in the sample. And the density of dentinal tubules increase as one approaches the dental pulp ( Garberoglio & Brannstrom,1976).

**Answer to comments on the aims:** The evidences supporting SM as a gold standard for dentin reactions to caries are typically those cited in the review paper by Kidd & Fejerskov (2004) (reference 25); to quote: references 6, 8 and 9, and Johnson *et al.*,(1969). They are not validation studies (i.e., accuracy is not provided), but qualitative studies where microradiographic images are reported without correction for heterogeneous illumination. Previous reports showed that translucent dentin could be either demineralization or sclerosis (references 11-14), and this fact means that the probability of finding similar results is high if SM and MR are compared many times. It is not excluded neither the possibility of finding translucent sclerotic dentin nor the possibility of finding a correlation between SM and MR when there is no translucent dentin. The study by Hintze et al. (reference 10) was performed with unerupted sound teeth, which lack translucent dentin in response to caries. Measurements of lesion size with SM exclude translucent dentin, resulting in underestimation of lesion size if part of the translucent dentin is demineralization. Translucent dentin comprises a significant part of dentin reactions to the carious process ( Bjørndal & Thylstrup,1995; Bjørndal *et al.*, 1999). On this basis, in the “Aims” we considered that further studies were needed to support SM as a gold standard for detecting dentin reactions to caries. The third aim does not include the test of the accuracy of SM. With all due respect, we think that the aims should be kept as they are.

Positive and negative predictive values are considered as elements of accuracy because they describe the performance of diagnostic tests and high results are indicative of good accuracy. In order to put this more clearly in the revised version, PPV and NPV will be specified as elements of accuracy in the aims.

**Answer to comments on materials and methods: **The statement “Examiner agreed on the final scores by consulting with each other” means that the final scores were given by consensus between examiners, just like was done with the visual diagnosis with ICDAS II described in “Diagnosis of occlusal caries lesions using ICDAS II”. When disagreement occurred, the examiners consulted each to reach a consensus. This is a common approach in the dental literature.

  

**Answer to comment on the statement ”… included in the sample … when detected using MR.”:** This statement was included to say that SM and MR scores were not the same. Histological sites were selected on the basis of MR images. In many cases, sites scored as normal dentin by SM, for example, were not scored as normal dentin by MR. This did not apply for all cases, as stated in the previous paragraph where it is written that “at least one dentin reaction type per sample was included where possible”. This was not possible for all cases. We recognize, however, that the statement is not clear, is misleading, and it will be changed in the revised version.

**Answer to comments on results: **We accept the suggestion of the referee on abbreviations. This modification will be included in the revised version.

**Answer to comments on Table 1:** Regarding the report that 10 teeth with ICDAS score 0 presented carious and sclerotic dentin, below follow reports of cases of occlusal surfaces with ICDAS score 0 that were scored by SM (with regard to the depth of the carious lesion) as having demineralization in the inner 50% of the enamel layer (E2) or in the outer 50% of the dentin layer (D1):

 

1. Ekstrand *et al.* (1997) reported 05 cases of occlusal caries with ICDAS score 0 and SM score E2 (see Table 8 in their paper);
2. Ekstrand *et al.* (1998) reported 01 case of occlusal caries with ICDAS score 0 and SM score E2 (see Table 4 in their paper);
3. Jablonski-Momeni *et al. *(2008) reported 02 cases of occlusal caries with ICDAS score 0 and SM score E2 (see Table 4 in their paper);
4. Braga *et al*. (2009) reported 01 case of occlusal caries with ICDAS score 0 and SM score E2 (see Table 4 in their paper);
5. Diniz *et al.* (2009) reported 06 cases of occlusal caries with ICDAS score 0 and SM score E2 and 01 case of ICDAS score 0 and SM score D1 (see Table 3 in their paper).

SM score E2 is important because it has been related to the occurrence of translucent dentin ( Bjørndal & Thylstrup,1995; Bjørndal *et al.*, 1999), and this later might be either carious or sclerotic (see our results and references 11-14), so that part or all of those cases with SM score E2 listed above could represent carious dentin. On this basis, the occurrence of carious and sclerotic dentin with ICDAS score 0 is not an unexpected result.

The totals of PLMQ and NFNBC differ because, as explained in the section “Polarized light microscopy in quinoline”, PLMQ is not used to detect sclerotic dentin. The total of PLMQ cases is the sum of carious plus normal dentin, while the total of NFNBC cases is the sum of carious plus normal plus sclerotic/translucent dentin. 

**Answer to comments on Figure 1: **The fact that figures are not explained following their sequence was intentional. The legend describes the figures following the sequence of the aspects of the sites 1-4 pointed in Fig.1A (wet SM). This way, emphasis in given to the main differences between the wet SM image (commonly used in the literature) and the others. Regarding the arrows, we had prepared a version with arrows in all figures but we thought that it would disturb free interpretation of some images by the reader. This is why arrows were included in half of the images. With all due respect, we still thing this way is the best one.

Answer to comments on Table 2: We accepted the suggestion of the reviewer and the title will be changed (in the revised version) to: “Table 2. PPV and NPV of SM and PLMQ for dentin reactions from all combinations of test and gold standard (MR) outcomes”.

**Answer to comments on Table 3:** (1-5) and (6) are the combinations described in the Material and Methods. In the revised version, we will explain them using a footnote in Table 3.
